# Effects of Coil Orientation on Motor Evoked Potentials From Orbicularis Oris

**DOI:** 10.3389/fnins.2018.00683

**Published:** 2018-11-13

**Authors:** Patti Adank, Dan Kennedy-Higgins, Gwijde Maegherman, Ricci Hannah, Helen E. Nuttall

**Affiliations:** ^1^Department of Speech, Hearing and Phonetic Sciences, University College London, London, United Kingdom; ^2^Sobell Department of Motor Neuroscience and Movement Disorders, Institute of Neurology, University College London, London, United Kingdom; ^3^Department of Psychology, Lancaster University, Lancaster, United Kingdom

**Keywords:** transcranial magnetic stimulation, motor cortex, facial muscle, hand muscle, motor evoked potentials, coil orientation

## Abstract

This study aimed to characterize effects of coil orientation on the size of Motor Evoked Potentials (MEPs) from both sides of Orbicularis Oris (OO) and both First Dorsal Interosseous (FDI) muscles, following stimulation to left lip and left hand Primary Motor Cortex. Using a 70 mm figure-of-eight coil, we collected MEPs from eight different orientations while recording from contralateral and ipsilateral OO and FDI using a monophasic pulse delivered at 120% active motor threshold. MEPs from OO were evoked consistently for six orientations for contralateral and ipsilateral sites. Contralateral orientations 0°, 45°, 90°, and 315° were found to best elicit OO MEPs with a likely cortical origin. The largest FDI MEPs were recorded for contralateral 45°, invoking a posterior–anterior (PA) current flow. Orientations traditionally used for FDI were also found to be suitable for eliciting OO MEPs. Individuals vary more in their optimal orientation for OO than for FDI. It is recommended that researchers iteratively probe several orientations when eliciting MEPs from OO. Several orientations likely induced direct activation of facial muscles.

## Introduction

**FIGURE 1:**
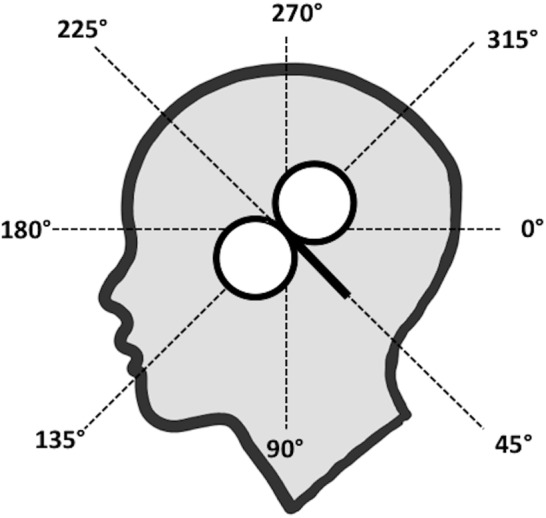
Eight coil orientations used in the lip and hand conditions. The intersection of the lines was placed on the subject’s hot spot for lip or hand M1 and the coil handle was aimed toward the angle tested (here: 45°).

Motor Evoked Potentials (MEPs) are crucial in characterizing motor system function in a variety of tasks (Pascual-Leone et al., 2000). Several consensus papers prescribe procedures for standardized MEP collection, specific to corticospinal (Rossini et al., 2015) and corticobulbar systems (Groppa et al., 2012). One well-known feature of hand muscle MEPs, innervated by the corticospinal tract, is their sensitivity to coil orientation and thus orientation of the induced current in the brain (Brasil-Neto et al., 1992; Mills et al., 1992; Werhahn et al., 1994). The recommended coil orientation for collecting MEPs from the hand is at an angle of 45° (cf. Figure 1) with respect to the sagittal plane, which induces a posterior–anterior (PA) current flow approximately perpendicular to the anterior wall of the central sulcus, as this evokes MEPs with the lowest stimulus intensities (Rossini et al., 2015).

When stimulating the corticospinal tract, two main patterns can be observed. First, low intensity PA currents preferentially evoke short-latency responses with the lowest thresholds, whilst anterior-posterior (AP) directed currents have a higher thresholds and preferentially evoke longer latency responses (Day et al., 1989; Sakai et al., 1997; Hannah and Rothwell, 2017). These differences are thought to reflect the recruitment of different sets of excitatory synaptic inputs to corticospinal axons, with different physiological (Hanajima et al., 1998; Hannah and Rothwell, 2017) and behavioral properties (Hamada et al., 2014; Hannah et al., 2017). Second, latero-medial (LM) oriented currents preferentially evoke the shortest latency responses likely resulting from direct activation of corticospinal axons, i.e., non-synaptic activation (D-waves), and are thus sub-optimal for evaluating changes in cortical excitability, since they effectively bypass cortical synapses. However, while the optimal coil orientation - and resulting induced current direction - for eliciting MEPs from muscles innervated by the corticospinal tract is fairly well-established, recommendations are less clear for facial muscles, innervated through the corticobulbar tract. A handful of studies report on the effect of coil orientation on MEPs elicited for various facial muscles, including Nasalis (Dubach et al., 2004), Masseter (Guggisberg et al., 2001), Depressor Anguli Oris and Depressor Labii Inferioris (Rodel et al., 1999), and muscles in the tongue (Murdoch et al., 2013), but results do not identify a single optimal orientation.

The Orbicularis Oris (OO) muscle is relevant to both clinical research, e.g., in Bell’s Palsy (Schriefer et al., 1988; Meyer et al., 1994), and cognitive neuroscience studies where OO MEPs have been recorded to investigate changes in motor cortex activity during speech perception (Fadiga et al., 2002; Roy et al., 2008; Murakami et al., 2011; Swaminathan et al., 2013; Nuttall et al., 2016, 2017). These studies induced a PA current flow by adopting the standard 45° downward pointing orientation recommended for hand MEP acquisition, despite the fact it has not been explicitly verified that the 45° orientation used for hand MEPs is also suitable for OO. From a practical point of view, using an optimized coil orientation for OO will allow clinical and cognitive neuroscience researchers to use lower stimulus intensities, thus potentially reducing subject dropout due to discomfort. Importantly, it may also help avoid the potential for direct activation of the corticobulbar axons (D-waves), which might otherwise confound the proper interrogation of the corticobulbar pathway.

Given the similar organization of the lip and hand motor cortex, a similar anisotropy of lip motor cortex responses to TMS might be assumed. Furthermore, it seems plausible that the optimal coil orientations for lip and hand are similar given their close location in the lip of pre-central gyrus / anterior bank of the central sulcus (Penfield and Boldrey, 1937) and the broadly similar orientation of the sulcus at each point. However, unlike in hand muscles, the location of the coil close to cranial nerves when eliciting facial MEPs lends to the possibility of directly activating motor nerves supplying facial muscles, to evoke M-waves, which could contaminate measurement of MEP amplitudes and latencies. The likelihood of directly activating facial muscles could depend on proximity of the coil to the nerve, and therefore on coil orientation. Dubach et al. (2004) reported direct innervation of ipsilateral and contralateral Nasalis muscle using surface electrodes; they report short latency responses (<7.5 ms) with a likely peripheral origin. These short-latency-responses were most commonly elicited for coil orientations 120° and 165°, i.e., orientations with the coil handle facing in an antero-ventral direction (i.e., directly over the subject’s temple), inducing approximately LM or AP currents. Given the proximity of the different facial regions in the cortex, we expected that responses evoked by TMS in OO might also consist of both peripherally- (M-waves) and cortically evoked (MEPs) responses, and that the presence of M-waves would vary per coil angle.

This study aimed to determine the effect of the direction of the induced current on the size and morphology of contra- and ipsilateral MEPs of the OO muscle by systematic manipulation of the coil orientation used to evoke MEPs. We measured MEPs evoked from OO and First Dorsal Interosseous (FDI), to enable comparison of OO results with the well-documented effect of current direction and coil orientation on FDI (Werhahn et al., 1994; Balslev et al., 2007), following stimulation of left lip and hand M1, respectively. We also examined the possibility of direct nerve innervation occurring for lip muscles: (i) by measuring OO MEPs from contra- and ipsilateral sites of OO, since M-waves would only be expected to be present on the side ipsilateral to stimulation; and (ii) by measuring the onset latencies of responses to determine their likely origin (i.e., cortical and synaptic versus peripheral and non-synaptic).

## Materials and Methods

### Subjects

We tested 16 subjects (seven males; average age: 30 years 2 months ± SD 6 years 11 months; age range: 24–47 years, 10RH, 6LH). Handedness was established via the Edinburgh handedness inventory (Oldfield, 1971). Data from one right-handed male subject were discarded due to a technical error during data collection. Subjects presented no TMS contraindications, and did not report any (history of) neurologic/psychiatric disease, or that they were under the effect of neuroactive drugs. All subjects had a minimum high school-level education, with the majority currently studying at University level. They were asked to not consume caffeine-containing drinks before the experiment and were all tested before noon. Experiments were undertaken with the understanding and written consent of each subject, according to University College London Research Ethics Committee (UREC).

### Transcranial Magnetic Stimulation

Monophasic single TMS pulses were generated by a Magstim 200^2^ unit and delivered by a 70 mm diameter figure-of-eight coil, connected through a BiStim^2^ module (Magstim, Dyfed, United Kingdom) set to simultaneous discharge mode (inter-pulse spacing of 0 ms). The coil was placed tangential to the skull such that the induced current flowed from posterior to anterior under the junction of the two wings of the figure-of-eight coil. The lip and hand areas of M1 and associated active motor threshold (aMT) for each muscle were found using the functional ‘hot spot’ localization method, cf. Nuttall et al. (2016). Active motor threshold was established using the standard 45 degree angle, during 20–30% background muscle contraction. The intensity resulting in 5 out of 10 TMS pulses yielding MEPs at or above the 0.2 mV criterion was taken as the (active) motor threshold. The intensity of the stimulator was then set to 120% of aMT for the stimulations applied during the experiment. The mean aMT used to elicit OO MEPs was 48.0% (± 5.6%), and 39.4% (± 6.4%) of the maximum possible intensity for FDI MEPs. Testing occurred at 120% of aMT; 57.6% (± 6.8%) for OO and at 47.4% (± 7.6%) for FDI.

### Electromyography

Electromyographic (EMG) activity was recorded from lip and hand areas using surface electrodes (Ag/AgCl; 10 -mm diameter) in a non-Faraday-caged, double-walled sound-attenuating booth. For the lips, electrodes were attached to OO on the both sides of the mouth, on the upper lip, approximately 5 mm from the vermillion border, orientated horizontally, in a bipolar, belly-belly montage, with electrodes placed at the left and right temples serving as a common ground. To stabilize background EMG activity, subjects were trained for approximately 5 min to produce a constant level of contraction (approximately 20% of maximum voluntary contraction) of the lip muscles by pursing, which was verified via visual feedback of the on-going EMG signal. For the recording of hand EMG, electrodes were attached in a tendon-belly montage with the active electrode placed on both FDI muscles, the reference electrode on the tendon of the same muscle, and a ground electrode on each wrist. Subjects were also trained to maintain a constant level of contraction of this muscle during the experimental recordings. Contraction of the lip and hand muscles also facilitates a lower motor threshold relative to when the muscle is at rest, enabling the use of lower levels of stimulation during the experiment. It is not straightforward to elicit MEPs from OO in the relaxed muscle because of the relatively high threshold. The raw EMG signal was amplified by a factor of 1000, band-pass filtered between 100–2000Hz, and sampled at 5000Hz online using a 1902 amplifier (Cambridge Electronic Design, Cambridge), and analog-to-digital converted using a Micro1401-3 unit (Cambridge Electronic Design, Cambridge). Continuous data were acquired and recorded using Spike2 software (version 8, Cambridge Electronic Design, Cambridge).

### Procedure

Following recommendation from Groppa et al. (2012) for exploratory non-clinical studies, we delivered TMS pulses to eight orientations: 0°, 45°, 90°, 135°, 180°, 225°, 270°, and 315° (Figure 1) in each subject for both sides of OO after identification of the hotspot and motor threshold for left lip M1. In a separate block, we delivered TMS pulses from the same orientations after stimulation of the hotspot for left hand M1 from left and right FDI. We therefore aimed to collect 480 MEPs over two channels per subject per muscle (960 in total). Subjects maintained 20% of maximal voluntary contraction in both hands, to make the results of the ipsilateral recordings comparable with both lip channels (it is not straightforward to relax only one side of OO). We counterbalanced the order in which hand and lip MEPs were collected across subjects. Within a lip or hand block, the coil rotation procedure was as follows. The subject wore an unmarked EEG cap. After localizing the hotspots for lip and hand M1, we attached a pre-constructed lattice made of adhesive tape aligned in a starburst pattern made of tape to the EEG cap. This lattice contained four intersecting guiding lines as in Figure 1. The intersection of the lines was placed on the hot spot for lip or hand M1, ensuring that the line between 180° and 0° in Figure 1 was approximately level with the parasagittal plane. The same lattice was used for all subjects. For each orientation, the handle of the coil was parallel with the guiding line for the target angle and the intersection between the two wings of the coil was on the intersection and on the hot spot. The coil aligned on the subject’s head using the tape guide, and 30 MEPs per orientation were collected. We used a relatively high number of MEPs compared to previous studies (Guggisberg et al., 2001; Dubach et al., 2004). A low number of trials per subject is associated with decreased statistical power and with inflated variability and noise. It has therefore been suggested to record at least 20–30 MEPs per condition in basic and clinical settings (Schmidt et al., 2009; Cuypers and Meesen, 2014; Goldsworthy et al., 2016). After completing testing for a single orientation, subjects were asked to report any muscle twitches and their level of comfort (1–7, 7 high comfort). The order for all eight angles was randomized as across subjects and the same order was used across lip and hand blocks. The duration of the session was between 2.5 and 3 h.

### Data Analysis

Individual EMG sweeps starting 40 ms before the TMS pulse and ending 1000 ms post-stimulation were exported offline from the recording software into Matlab, where mean MEPs were calculated for the two channels per TMS target, orientation, and subject. Individual averages were rectified and the integrated area under the curve (AUC) of the rectified EMG signal of each individual mean MEP was calculated as millivolts over millisecond (mV⋅ms). We chose to measure AUC instead of p-p - a measure commonly used for hand MEPs - as OO MEPs tend to consist of multiple peaks, in contrast with hand MEPs, which tend to consist of two successive midline deflections (peak and trough) (Adank et al., 2016). It is not straightforward to measure p-p amplitudes when successive peaks are present, and the amplitude of lip MEPs can be underestimated if p-p is used as successive peaks in an OO MEP complex would be excluded from final amplitude measurements. For lip MEPs, AUC was automatically computed from 8 to 35 ms post-TMS, and for hand AUC was computed from 13 to 40 ms post-TMS.

To confirm that the AUCs reflected cortically generated MEPs, and not the activity resulting from direct innervations of facial nerves, we examined the data taking into consideration latency. The analysis of latencies was conducted by hand using visual inspection, in a separate process from the AUC analysis (which was conducted using Matlab scripts). Short latencies suggest that the MEPs originated via direct innervation of facial muscles (cf. Dubach et al., 2004). We split the OO data into short latency (<7.5 ms) and middle latency (≥7.5 ms) responses. Average MEPs from ipsi- or contralateral muscles and for the eight orientation were compared using a non-parametric ANOVA that allowed for missing values, the Skillings-Mack test (Chatfield and Mander, 2009), for both effectors (hand or lip) separately.

## Results

### Lip

Mean AUCs for OO are reported in Table 1 and latencies in Table 2, respectively. MEPs from OO for 135° were not recorded for a male left-handed subject due to a technical error. Seventeen of 240 average MEPs were classified as outliers (>1.5 the interquartile range) and excluded from further analysis.

**Table 1:** Average Area Under the Curve (AUC) in mV⋅ms, plus standard deviations (SD), and number of subjects (N) contributing to the average, for Orbicularis Oris (OO) and First Dorsal Interosseous (FDI) muscles per coil orientation.

OO	Contralateral			Ipsilateral		
	AUC	SD	N	AUC	SD	N
0°	8.6	7.0	15	3.3	1.1	12
45°	8.2	5.4	14	3.5	1.2	12
90°	6.3	3.5	15	3.4	2.0	14
135°	6.8	3.2	15	6.2	5.7	13
180°	6.3	3.6	15	9.5	8.7	14
225°	2.7	1.4	15	2.2	0.7	15
270°	2.9	1.5	14	2.0	0.9	13
315°	7.2	5.7	15	3.3	1.7	11
**FDI**						
0°	21.7	15.4	15	2.5	1.3	14
45°	36.3	17.2	15	2.8	1.8	15
90°	10.9	9.2	13	2.0	1.3	14
135°	5.9	6.1	15	2.9	1.7	15
180°	5.9	7.7	15	2.7	1.3	15
225°	4.2	1.5	12	2.3	1.2	13
270°	4.8	3.5	13	2.8	1.3	14
315°	5.7	5.0	14	2.7	1.1	15

**Table 2:** Average response latency duration in milliseconds, plus standard deviations (SD), and number of subjects (N) contributing to the average, for Orbicularis Oris (OO) and First Dorsal Interosseus (FDI) muscles.

OO	Contralateral			Ipsilateral		
	Latency	SD	N	Latency	SD	N
0°	9.6	1.1	6	–	–	–
45°	9.7	1.2	7	6.3	–	1
90°	8.9	0.7	6	8.3	2.8	2
135°	7.4	1.4	9	6.9	1.7	4
180°	6.7	0.8	8	6.6	0.7	7
225°	–	–	–	–	–	–
270°	–	–	–	–	–	–
315°	9.1	2.5	6.0	–	–	–
**FDI**						
0°	20.8	2.1	10	–	–	–
45°	20.8	1.9	14	6.3	–	1
90°	20.8	1.5	3	8.3	2.8	2
135°	21.5	–	1	6.9	1.7	4
180°	27.0	–	1	6.6	0.7	7
225°	–	–	–	–	–	–
270°	–	–	–	–	–	–
315°	22.0	3.5	2	–	–	–

**FIGURE 2:**
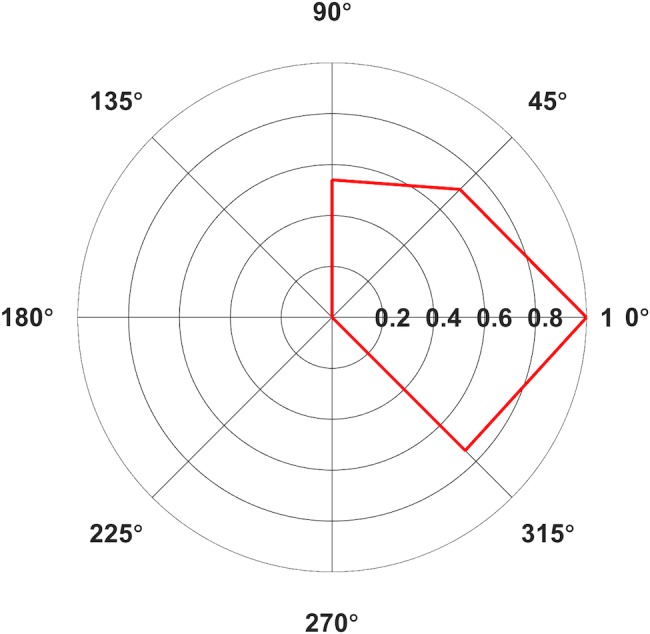
Polar plot of average Area Under the Curve (AUC) elicited from contralateral (A) Orbicularis Oris (OO) in mV⋅ms for Middle Latency MEPs only. Only average values with >5 contributing subjects are included. Values normalized relative to the largest value, set to 1.

**FIGURE 3:**
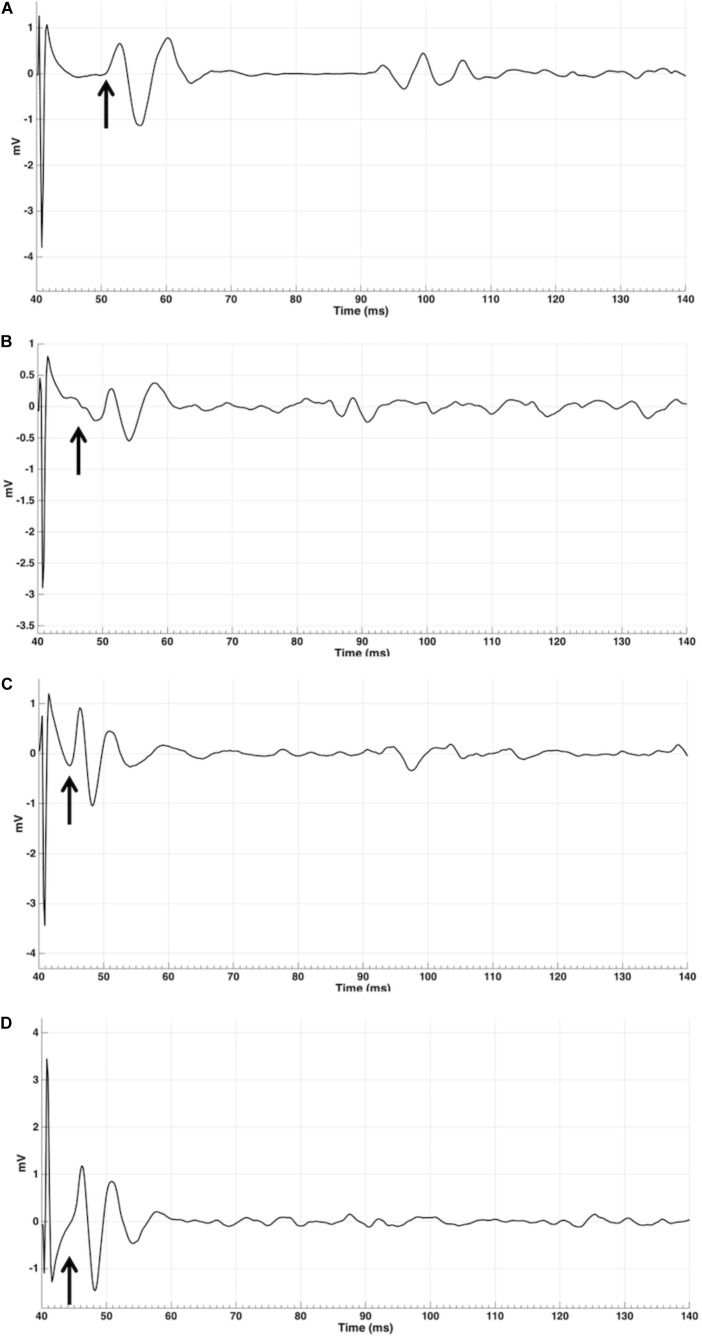
MEP EMG traces for a single MEP for subject 9 for four orientations: contralateral 45 **(A)**, contralateral 135 **(B)**, ipsilateral 0 **(C)**, and 180 **(D)** in mV. Arrows indicates approximate start of MEP, measured from TMS pulse at 40 ms into the trial.

Latencies (Table 2) were measured for at least 5 out of 15 subjects for contralateral OO in 0°, 45°, 90°, 180°, and 315°, in ipsilateral OO for 180°. Middle latency responses were recorded for contralateral 0°, 45°, 90°, and 315° only. Short latencies were found for 135° and 180° (Figure 2). Figure 3 shows raw MEPs for one subject collected from contra- and ipsilateral orientations to illustrate effects of coil orientation on latency. Note the short latency for response collected from 180° contralateral and 135° ipsilateral orientations compared to the latency for 45° contralateral. Moreover, subjects reported muscles twitches on fifty occasions: in jaw (30), eye (11), forehead (2), face (2), neck (3), lip (1), or nose (1). Note that the presence of a muscle twitch does not automatically imply direct nerve activation, but that muscle twitches could also result from a twitch in the target muscle. The majority of facial twitches were reported for 180° (11), followed by 0° (9), 270° (7), and 45° (6). Note that TMS to lip M1 generally does not result in noticeable twitches in OO, and it was reported only once throughout the entire experiment. The number of reported twitches for lip was higher than for the hand condition (15), presumably as on average higher stimulator output was used in the lip condition (48% versus 38% for the hand condition). Also, a more ventral, and for most subjects also more anterior, placement of the coil was used to target lip M1, and the coil was therefore closer to superior branches of the facial nerve (i.e., the temporal and zygomatic branches).

Based on the measurements of latencies, it appears that MEPs with a cortical (synaptic) origin were plausibly invoked predominantly in contralateral sites with the coil handle positioned at 315°, 0°, 45°, and 90° (Figure 1), and in two subjects in 90° ipsilaterally. MEPs generated in all ipsilateral orientations, potentially with the exception of 90°, and contralateral orientations 135°, 180°, 225°, and 270° likely had a peripheral origin (but we cannot exclude the possibility that these responses were the result of direct activations of contralateral corticobulbar) and may have resulted from direct stimulation of facial motor nerves. We included the averages for contralateral orientations 0°, 45°, 90°, and 315° in the Skillings-Mack test (contralateral 135° was not included as the test does not allow levels with fewer than three observations) and found no difference in AUC [*t*(3) = 5.0589, *p* = 0.1675]. This indicated that these four orientations resulted in comparable AUCs. However, it seems like contralateral 0° and 45° are optimal for evoking OO MEPs, as this orientation resulted in the highest proportion of middle latency MEPs (Table 2) for a majority of subjects.

### Hand

**FIGURE 4:**
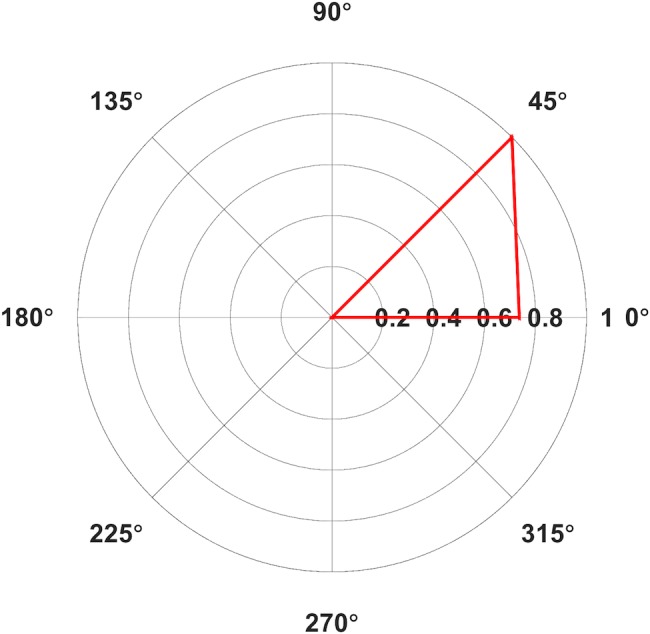
Polar plot of average Area Under the Curve (AUC) elicited from contralateral (A) First Dorsal Interosseous (FDI) in mV⋅ms for Middle Latency MEPs only. Only average values with >5 contributing subjects are included. Values normalized relative to the largest value, set to 1.

Mean AUCs for FDI are reported in Table 1 and latencies in Table 2. One subject erroneously did not contract her right hand during collection of bilateral FDI MEPs. Fifteen out of 240 average MEPs were classified as outliers and were excluded from further analysis. Subjects reported muscles twitches in their hand (7), jaw (2), eye (2), face (2), nose (1), or neck (1) on 15 occasions. Facial twitches were reported most often for 135° (4), followed by 180° (3). We compared the average AUC values between contralateral orientations 0° and 45° in the Skillings-Mack test and found a significant difference in AUC [*t*(1) = 4, *p* = 0.0455], with larger AUCs for 45° than for 0° (Figure 4).

## Discussion

Our results replicated previous findings by showing that 45° was the optimal orientation for FDI MEPs. Middle latency (∼20 ms) responses were found only for contralateral 45° and 0°. MEPs recorded from ipsilateral FDI had short latencies (<10 ms). The results for FDI replicate findings previously reported for studies investigating the effect of coil orientation (Pascual-Leone et al., 1994; Werhahn et al., 1994; Balslev et al., 2007).

For OO, after controlling for the presence of potential non-cortical responses, MEPs could be evoked across a broader range of contralateral angles, 0°, 45°, 90°, and 315°, and these were also the only orientations that generated middle latency MEPs in the majority of subjects. Ipsilateral responses were rare and tended to include short-latency responses that are most likely due to direct activation of facial nerves. Specifically, the OO results showed the largest MEP amplitudes for contralateral orientations. It is not clear why the range of orientations (and associated currents) is wider for OO than for FDI. Speculatively, Moreover, the optimal orientation for OO varies across individuals and that optimal MEPs for an individual might be collected at 0°, 45°, 90°, or 315°. As was the case for previous results on the effect of coil orientation on elicitation of MEPs in other facial muscles (Rodel et al., 1999; Pilurzi et al., 2013), we measured ipsilateral OO MEPS in several subjects, although the low amplitudes and short latencies found for the majority of subjects indicate that these were most likely M-waves. Rodel et al. (1999) report ipsilateral MEPs in OO for all their subjects and also report slightly larger MEP amplitudes for ipsilateral MEPs for a subset of their tested coil positions relative to vertex, while Pilurzi et al. (2013) report ipsilateral MEPs for 14 out of 18 subjects for the Depressor Anguli Oris muscle. Moreover, Triggs et al. (2005) collected contra and ipsilateral MEPs from OO after stimulating left and right M1. Ipsilateral MEPs were larger than contralateral MEPs after TMS to left and right lip M1 in a subset of Triggs et al.’s subjects (14 of 42), in line with our results for OO from several ipsilateral orientations (Table 1). Ipsilateral MEPs in OO may originate from cortical sources from I-waves generated by pyramidal neurons in M1, or through direct innervation of the facial nerves, as discussed earlier. Even though OO is the only median facial muscle, it does not seem likely that the recorded MEPs were due to action potentials traveling across muscle fibers between both sides of the lips. When muscular fibers in OO were directly stimulated innervated via the facial nerve ipsilaterally have been reported to cross the midline for only a few millimeters (Trojaborg, 1977). Midline crossing of motor axons in facial nerves can occur in pathological conditions such as complete unilateral facial palsy (Gilhuis et al., 2001). The subjects reported the highest proportion of facial (jaw) twitches for 180°, an orientation in which the coil handle is positioned (Figure 1) so that it seems feasible that we might have directly stimulated upper branches of the facial nerve. However, lower face twitches were also reported for other orientations (0°, 270°, and 45°), so it seems implausible that orientation can be directly linked to facial nerve innervation. Our data does not allow for conclusive elimination of the possibility that ipsilateral MEPs were evoked by direct stimulation of the ipsilateral facial nerve. Moreover, according to Ziemann et al. (1999) pathways for ipsilateral and contralateral MEPs can be dissociated. It is possible to use TMS to activate different types of corticofugal motor fibers, including the fast-conducting crossed corticomotoneuronal. Ipsilateral oligosynaptic pathway, such as a corticoreticulospinal or a corticopropriospinal projection, have also been suggested as possible routes for the ipsilateral MEP. It might have been the case that the ipsilateral MEPs in our paper originate from a fast-conducting crossed corticomotoneuronal pathway. Nevertheless, to more conclusively clarify the origin of ipsilateral MEPs, and of MEPs evoked from contralateral orientations 135°, 180°, and 270° it would be useful, to employ a paired-pulse protocol such as SICI (Short-Interval Intra-cortical Inhibition) (Kujirai et al., 1993). SICI is present in lower facial muscles, as demonstrated by Pilurzi et al. (2013).

## Conclusion

Our results for FDI and OO replicate and extend results of previous studies investigating optimal coil orientation in hand (Werhahn et al., 1994; Balslev et al., 2007). Our results indicate that coil orientations and associated induced current directions previously reported for muscles of the corticospinal tract, particularly FDI, were also appropriate for OO, a muscle innervated by the corticobulbar tract. However, the analysis pointed toward more variability in optimal orientation across subjects with respect to the optimal coil orientation for eliciting the largest MEPs, so we recommend examining a range of coil orientations spaced between 315° and 90° contralaterally when collecting MEPs from the active muscle. Specifically, we suggest taking an individualized approach to determining the optimal rotation for lip muscles. We suggest to systematically probe up to four orientations (0°, 45°, 90°, and 315°) for OO. This could be achieved by in a first step determining aMT for 45°, for 0°, 90°, and 315°. Next, a series of MEPs could be collected at a supra-threshold intensity (e.g., ≥120% aMT) from each orientation to see which produces the largest responses that can be verified as MEPs based on the presence of a latency >7.5 ms and a silent period following the response.

## Ethics Statement

This study was carried out in accordance with the recommendations of UCL Research Ethics Committee (UREC, #0599.001). The protocol was approved by UREC. All subjects gave written informed consent in accordance with the Declaration of Helsinki.

## Author Contributions

PA designed the study, collected and analyzed the data, and wrote the paper. DK-H and GM assisted in data collection and writing the paper. RH and HN assisted in data analysis and writing the paper.

## Conflict of Interest Statement

The authors declare that the research was conducted in the absence of any commercial or financial relationships that could be construed as a potential conflict of interest.
